# Effect of Silicate Additive on Structural and Electrical Properties of Germanium Nanowires Formed by Electrochemical Reduction from Aqueous Solutions

**DOI:** 10.3390/nano12162884

**Published:** 2022-08-22

**Authors:** Anna S. Eremina, Ilya M. Gavrilin, Nikolay S. Pokryshkin, Alexander Yu. Kharin, Alexander V. Syuy, Valentin S. Volkov, Valery G. Yakunin, Sergei S. Bubenov, Sergey G. Dorofeev, Sergey A. Gavrilov, Victor Yu. Timoshenko

**Affiliations:** 1 Phys-Bio Institute, National Research Nuclear University MEPhI, 115409 Moscow, Russia; 2 Frumkin Institute of Physical Chemistry and Electrochemistry of RAS, 119071 Moscow, Russia; 3 Faculty of Physics, Lomonosov Moscow State University, 119234 Moscow, Russia; 4 Center for Photonics and 2D Materials, Moscow Institute of Physics and Technology, 141700 Dolgoprudny, Russia; 5 Faculty of Chemistry, Lomonosov Moscow State University, 119234 Moscow, Russia; 6 Institute of Advanced Materials and Technologies, National Research University of Electronic Technology—MIET, 124498 Zelenograd, Russia

**Keywords:** nanowires, germanium, silicon, electrodeposition, Raman spectroscopy, electrical conductivity, gas sensorics

## Abstract

Layers of germanium (Ge) nanowires (NWs) on titanium foils were grown by metal-assisted electrochemical reduction of germanium oxide in aqueous electrolytes based on germanium oxide without and with addition of sodium silicate. Structural properties and composition of Ge NWs were studied by means of the scanning and transmission electron microscopy, X-ray photoelectron spectroscopy, X-ray diffraction, and Raman spectroscopy. When sodium silicate was added to the electrolyte, Ge NWs consisted of 1–2 at.% of silicon (Si) and exhibited smaller mean diameter and improved crystallinity. Additionally, samples of Ge NW films were prepared by ultrasonic removal of Ge NWs from titanium foils followed with redeposition on corundum substrates with platinum electrodes. The electrical conductivity of Ge NW films was studied at different temperatures from 25 to 300 °C and an effect of the silicon impurity on the thermally activated electrical conductivity was revealed. Furthermore, the electrical conductivity of Ge NW films on corundum substrates exhibited a strong sensor response on the presence of saturated vapors of different liquids (water, acetone, ethanol, and isopropanol) in air and the response was dependent on the presence of Si impurities in the nanowires. The results obtained indicate the possibility of controlling the structure and electrical properties of Ge NWs by introducing silicate additives during their formation, which is of interest for applications in printed electronics and molecular sensorics.

## 1. Introduction

Germanium (Ge) nanowires (NWs) have a wide range of applications due to their electrical and optical characteristics [1]. Interest in Ge NWs is primarily due to the small band gap of crystalline Ge (c-Ge), the high mobility of charge carriers, and the low effective mass of an electron. Such properties of c-Ge are also retained in Ge NWs [2]. It was demonstrated that germanium nanostructures can be used in lithium- and sodium-ion batteries [3,4,5,6,7,8,9], thermoelectric converters [10,11,12], and photodetectors [13,14,15,16,17].

Semiconductor Ge NWs are most often obtained by vapor phase deposition, for example, by the vapor–liquid–solid (VLS) method, which is usually realized at high temperatures above 300–400 °C that can limit the choice of substrate materials [18,19,20,21,22,23,24,25]. While attempts are steadily being made to lower the VLS synthesis temperatures below 300 °C, the room temperature VLS synthesis of Ge NWs seems to be unrealizable [26]. An alternative approach is based on the electrochemical deposition of Ge NWs from aqueous solutions at room temperatures [27,28,29,30]. The corresponding growth mechanism, which has been referred to in the literature as electrochemical liquid–liquid–solid (ec-LLS), is similar to the VLS method. The idea behind the ec-LLS method is to use low-melting metal particles, such as mercury, gallium, and indium. The metal serves as an electrode for the reduction of Ge containing ions to germanium in the atomic state, with its subsequent dissolution and the formation of a eutectic melt with the metal. The continuous reaction of cathodic reduction provides concentration supersaturation of the melt with germanium, resulting in crystallization of Ge at the interface with the substrate.

This method is probably one of the simplest and most versatile ways to produce NWs, since nanostructures can be produced without complex and expensive equipment or high-vacuum systems. The advantages of the presented method are also low operating temperatures (most often the process takes place at room temperature), ease of scaling, customization, and environmental friendliness. Various impurities in the electrolyte for the ec-LLS process should influence the growth of Ge NWs, and as a result their properties can be controlled. On the one hand, the incorporation of silicon (Si) into the germanium lattice can result in the creation of various defects, which can decrease the carrier mobility and electrical conductivity of Ge NW structures. On the other hand, Si atoms can play the role of oxygen scavengers that will result in creation of silicon oxide clusters, which can stabilize the electrical properties of Ge-Si NWs in air.

In our work, Ge NWs without and with the addition of a Si-containing precursor were prepared by using a process of electrochemical reduction from aqueous solutions of germanium oxide. The prepared samples were investigated by different methods and tested as a material for potential sensor applications. The novelty of our approach consists of both the possibility of controlling the structural and electrical properties of Ge NWs by introducing Si-based impurities and demonstrations of a gentle transfer of the prepared NWs on another substrate that should expand possible applications of the former in printed electronics and sensorics.

## 2. Materials and Methods

Arrays of Ge NWs were obtained by cathodic electrochemical deposition on a titanium (Ti) substrate using indium nanoparticles as germanium reduction centers. The technique for forming an array of spherical metal particles is described in Ref. [31]. Electrodeposition was carried out in a two-electrode cell, the Ti foil was pressed against the cell, and a platinum wire was used as the counter electrode. The solution contained germanium oxide (IV) GeO_2_ (0.05 M), potassium sulfate K_2_SO_4_ (0.5 M) as an indifferent electrolyte, succinic acid C_4_H_6_O_4_ (0.5 M) as a buffer additive, and sodium silicate Na_2_SiO_3_ (0.01 M/0.02 M/0.03 M) as an additive. Samples of germanium nanowires prepared with a silicate addition were labeled as Ge-Si NWs. The pH of the solution was adjusted to 6.5 by adding NaOH. The deposition on the Ti foil substrate decorated with small indium nanoparticles was carried out at a current density of 0.3 mA/cm^2^ (U = 6.0 V) for 30 min at room temperature. The samples were washed in deionized water and dried in air [28]. Additionally, Ge and Ge-Si NWs were transferred into a solution with isopropyl alcohol, separating the NW layers in an ultrasonic bath for 5 min at room temperature. The prepared suspensions of Ge and Ge-Si NWs were used to form thin films on corundum substrates for electrical measurements.

Images of Ge NWs and Ge-Si NWs were obtained by scanning electron microscopy (SEM) on a MAIA 3 Tescan instrument at an accelerating voltage of 10, 15, and 20 kV, and high-resolution transmission electron microscopy (TEM) using a Jeol JEM-2100 microscope, with a resolution of 0.19 nm.

The elemental composition and chemical state of NW elements were studied by means of X-ray photoelectron spectroscopy (XPS) with a PHI5500 Versa Probe II Instrument. The concentrations were determined by the method of relative elemental sensitivity factors from the measured integral intensities of the following lines: C1s, O1s, Ge3d, In3d3, Na1s, K2p, Si2p, Cl2s, and F1s. The binding energies of the photoelectron lines were determined from high-resolution spectra recorded at an analyzer transmission energy of 23.5 eV and a data collection density of 0.2 eV/step. To obtain the concentration profiles of the Ge-Si NW sample, we used argon ion sputtering of the surface. This was performed using 2 keV Ar^+^ ions with the raster size of 2 × 2 mm^2^. The sputtering rate on SiO_2_ in this mode is about 9 nm/min; according to the reference data, the etching rate of Ge should be 2.25 times higher, i.e., about 20 nm/min. XPS data were analyzed with PHI Multipak software.

The phase composition and crystallinity of the prepared samples were studied using X-ray diffraction analysis (XRD) using a DR-02 “RADIAN” diffractometer. Recording conditions were: radiation from a copper anode (Cu–Kα) with a nickel filter, scanning step 0.08°, exposure time 12 s/point, current 5 mA, and voltage 25 kV.

The structural properties of Ge and Ge-Si NWs were also analyzed by means of Raman spectroscopy with a Horiba Jobin Yvon Lab RAM HR Visible high-resolution spectrometer. Spectra recording conditions were: monochromatic grating: 1200 f/mm, confocal hole 300 µm, slit 100 µm, exposure time 60 s, ND filter 0.4 dB, and magnification ×10. The size of the analyzed region of the sample was 2.5 μm. The spectra were recorded at room temperature.

Electrophysical measurements in ultrahigh vacuum (2–5∙10^−9^ Torr) were carried out in a chamber of the Auger spectrometer JAMP-10 (JEOL). For the study, NWs were deposited in the form of films on corundum chip substrates. The substrates were equipped with platinum contacts and a platinum heater on the reverse side, which simultaneously served as a temperature sensor. The electrical conductivity was recorded using a P-8 Nano potentiostat (Elins Electrochem. Instr., Chernogolovka, Russia). The heater was controlled using a P-30J potentiostat (Elins Electrochem. Instr., Chernogolovka, Russia). In experiments on the temperature dependence of conductivity, a triangular voltage sweep of up to 2 V was applied to the heater, while the voltage across the sample was constant, usually 10 V, but in the case of samples with high conductivity, 1 V, in order to avoid heating the sample with its own current. When recording the I–V characteristics, a triangular signal was applied to the sample; the voltage range during recording of the characteristics was typically ±15 V.

Ge NWs on corundum chips were tested as a sensor on vapors of different liquids (water, ethanol, isopropanol, and acetone) in air at room temperature. The sensor response was measured as a relative change in the electrical resistance of Ge NWs at an applied voltage of 3 V. The chip with Ge NWs was placed into a hermetic glass vessel of 20 mL, which could be filled with saturated vapors of investigated liquids at 25 °C. Prior to the sensor tests, the samples were preheated at 120 °C for 30 min in air, and then they cooled down to 25 °C. The electrical resistance was measured 5 min after vapor admission.

## 3. Results and Discussion

### 3.1. Scanning Electron Microscopy

**Figure 1:**
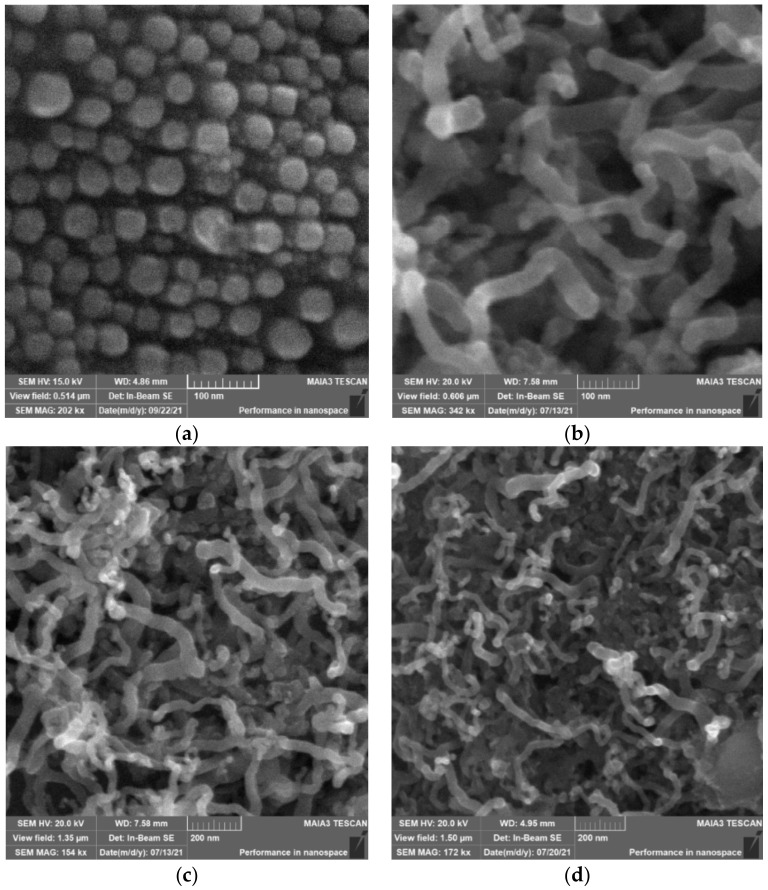
SEM images: (**a**) Indium nanoparticles deposited on Ti foil; (**b**,**c**) Ge NWs; (**d**) Ge-Si NWs (0.01 M Na_2_SiO_3_).

Figure 1a shows an SEM image of the surface of the Ti foil with deposited indium nanoparticles. It can be seen that the average diameter of nanoparticles is about 40 nm. Figure 1b,c show, at different scales, the formed Ge NWs, which have a curved shape, an average diameter of about 40 nm, and a length of 300 to 600 nm. Compared to Ge NWs, Ge-Si NWs obtained in a solution with a Na_2_SiO_3_ content of 0.01 M (Figure 1d) did not change in morphology, but the average diameter decreased to 30 nm. With an increase in the content of silicate to 0.03 M, the average diameter of the NWs remained at the same level. It can be assumed that the addition of silicate to the electrolyte limits the growth of NWs due, for example, to slower diffusion of germanic acid ions to indium nanoparticles, which makes the growth of NWs preferable for smaller metal nanoparticles.

### 3.2. Transmission Electron Microscopy

**Figure 2:**
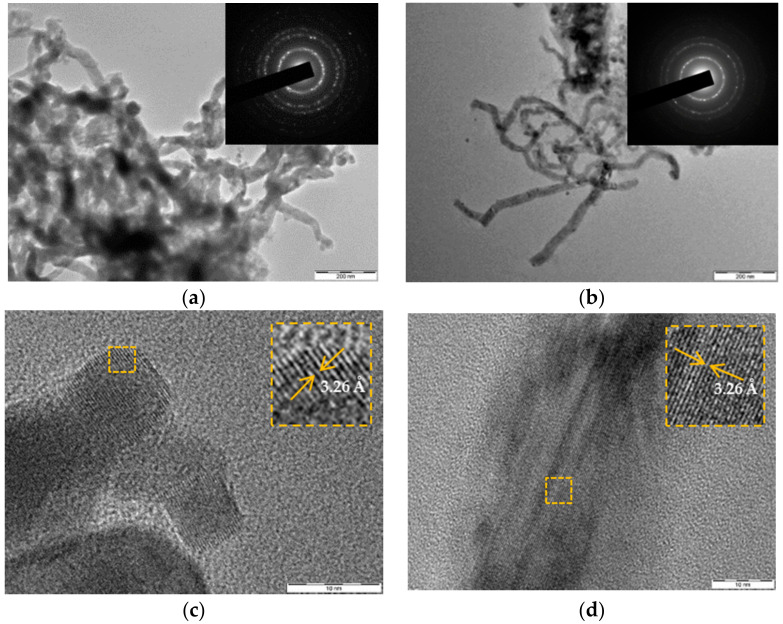
TEM images: (**a**) Ge NWs and (**b**) Ge-Si NWs, insets show corresponding electron diffraction patterns; high-resolution TEM patterns: (**c**) Ge NWs and (**d**) Ge-Si NWs, where insets show the corresponding magnified areas of the images, indicating the interplane atomic spacing.

Figure 2 shows TEM images and electron diffraction patterns for Ge and Ge-Si NWs dried from the prepared suspension. The cross-sectional size of NWs is about 30 and 40nm for Ge-Si and Ge samples, respectively, which agrees with the SEM data discussed above (see Figure 1). The electron diffraction patterns (insets in Figure 2a,b) show bright spots related to germanium nanocrystals with a certain orientation of atomic planes. For the Ge NW sample, the high-resolution TEM image shows nanocrystals with sizes of 6–10 nm (Figure 2c). Nanocrystals in Ge-Si samples have an elongated shape with a transverse size of 20–30 nm (Figure 2d). The measured interplanar spacing agrees with the known value d(111) = 3.26 Å for c-Ge.

### 3.3. X-ray Photoelectron Spectroscopy

**Figure 3:**
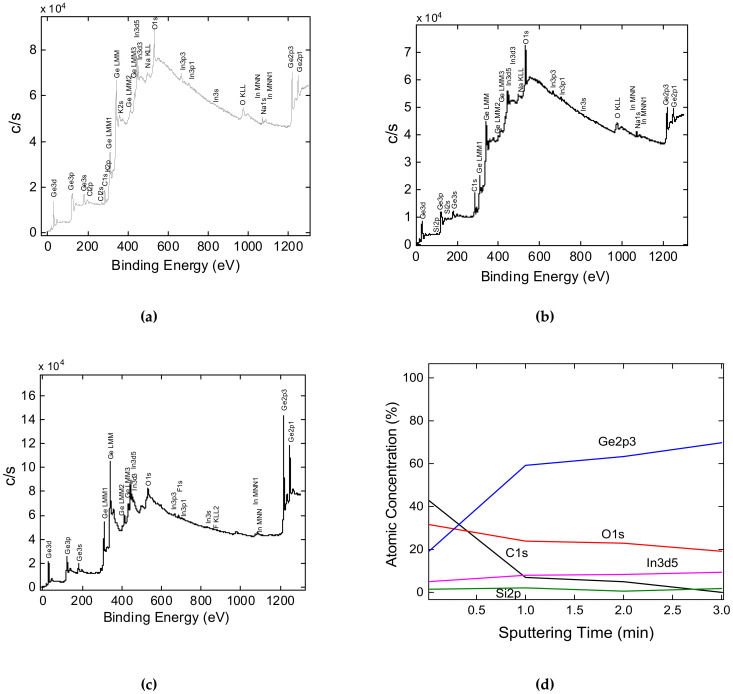
Overview spectra: (**a**) Ge NWs; (**b**) Ge-Si NWs; (**c**) Ge-Si NW after 3 min of sputtering; (**d**) Ge-Si NWs concentration profile.

Figure 3 shows survey XPS spectra of the investigated samples. The element concentrations are shown in Table 1. According to the XPS results, silicon was found in the Ge-Si NW sample, and an increase in the O/Ge and K/Ge ratios relative to the Ge NW sample was detected. After 3 min of Ar ion sputtering of the Ge-Si NW sample, no carbon impurity was detected (Figure 3d). The O/Ge ratio decreased by 3 times, and the Si/Ge ratio decreased by 4 times for the samples after Ar sputtering (Table 1).

**Figure 4:**
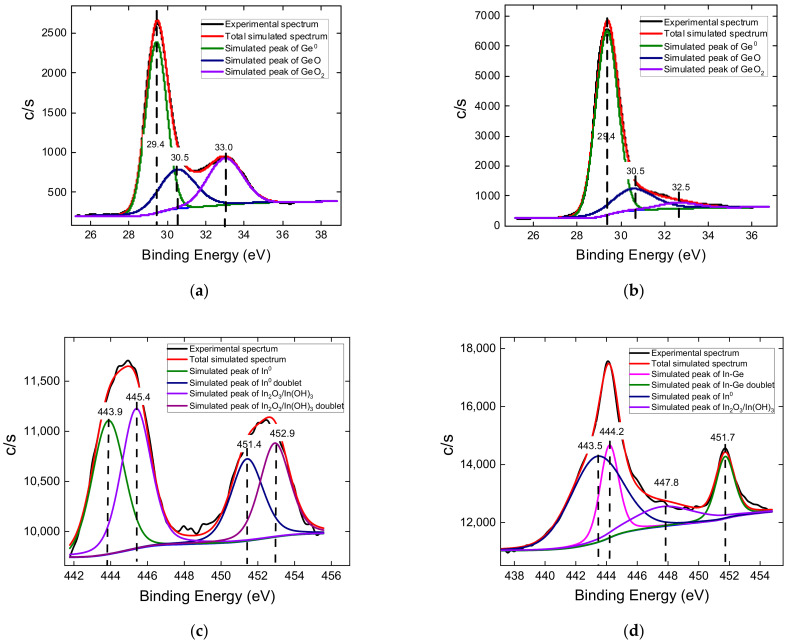

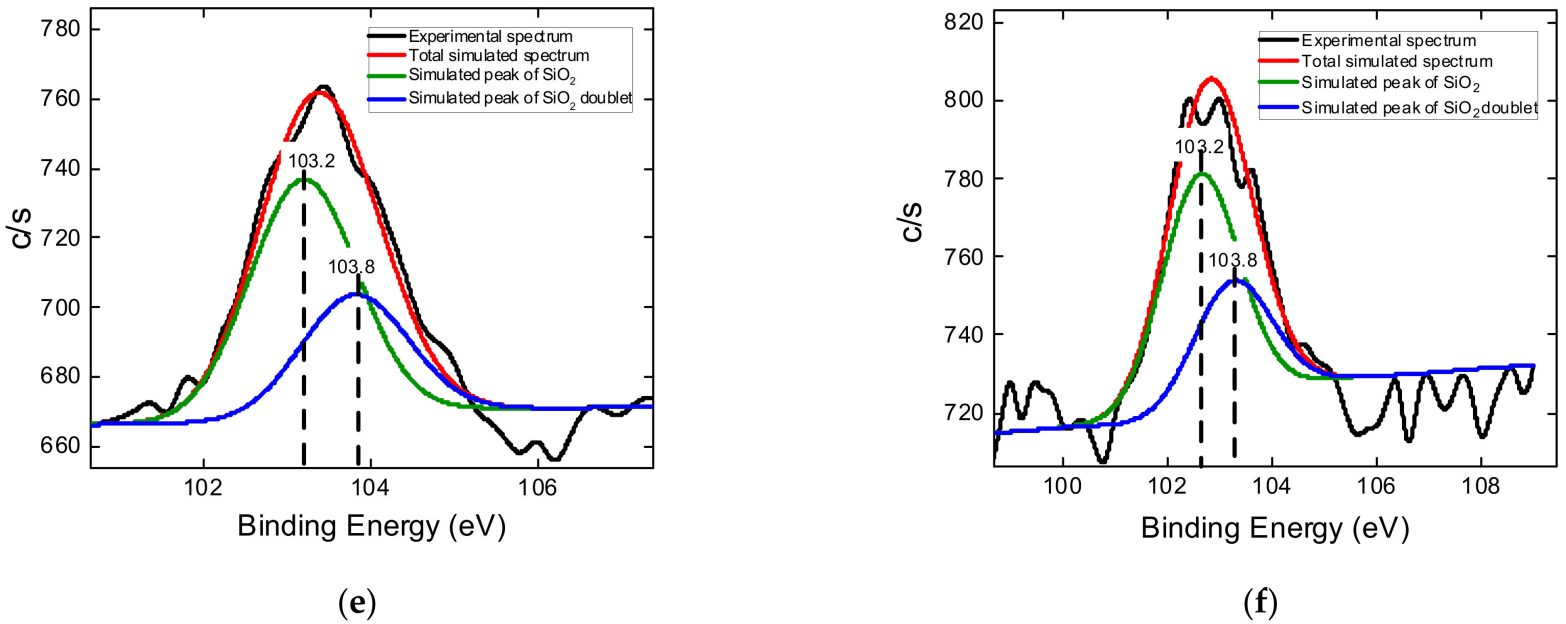
XPS spectra of Ge-Si NWs samples in the following regions: (**a**) Ge3d; (**b**) Ge3d after 3 min sputtering; (**c**) In3d; (**d**) In3d after 3 min sputtering; (**e**) Si2p; (**f**) Si2p after 3 min sputtering. Dashed lines indicate corresponding binding energies.

High-resolution XPS spectra are presented in Figure 4. An analysis of the spectra of germanium and indium on the original surface (Figure 4a,c) showed that both elements are partially oxidized. In the spectra of Ge3d, three peaks were distinguished on the initial surface: peak 1 at 29.4 eV from neutral germanium Ge^0^, peak 2 at 30.5 eV from GeO, and peak 3 at 33.0 eV from GeO_2_. After sputtering, the proportion of oxidized germanium decreased markedly (Figure 4b). In the In3d spectra, two doublets were distinguished on the initial surface: a 1-1’ doublet from metallic indium and a 2-2’ doublet from indium oxides/hydroxides In_2_O_3_/In(OH)_3_. After sputtering (Figure 4d), In was detected, which is located in the Ge NWs, so the position of its spectrum is slightly shifted due to the shift in the electron density from In atoms to Ge atoms. The data for Ge3d and In3d spectrums are summarized in Table 2.

The XPS spectrum of Ge-Si NWs in the Si2p region (Figure 4e) is represented by one Si2p3/2-Si2p1/2 doublet, and the E_b_ of the 2p3/2 peak is 103.2 eV, which corresponds to SiO_2_, taking into account the scale adjustment for the Ge3d peak from Ge^0^. Additionally, after etching, the position and intensity of the Si peaks almost did not change. This fact indicates the presence of Si atoms in the bulk of the Ge NWs rather than on their surface.

### 3.4. X-ray Diffraction Analysis

**Figure 5:**
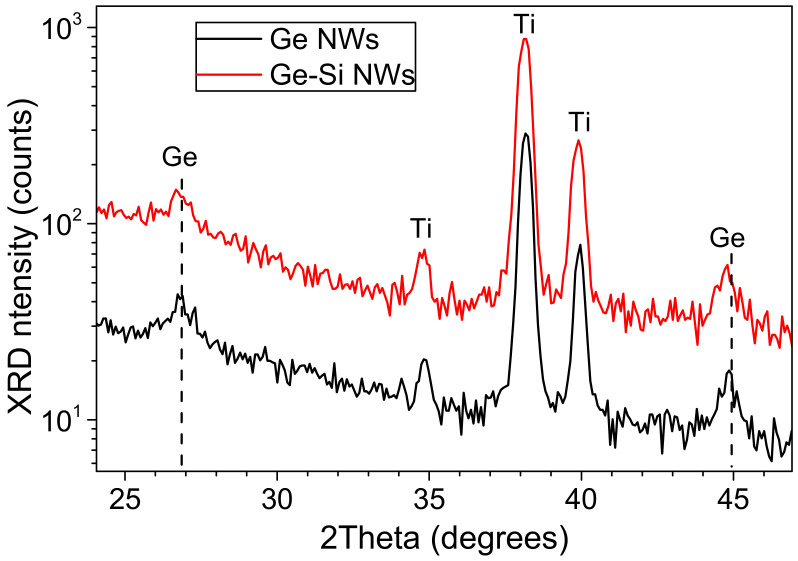
XRD angular spectra for Ge NWs (black line) and Ge-Si NWs (red line). The vertical dashed lines indicate the peak maxima of crystalline Ge and the peaks of Ti substrate are marked as “Ti”.

**Figure 6:**
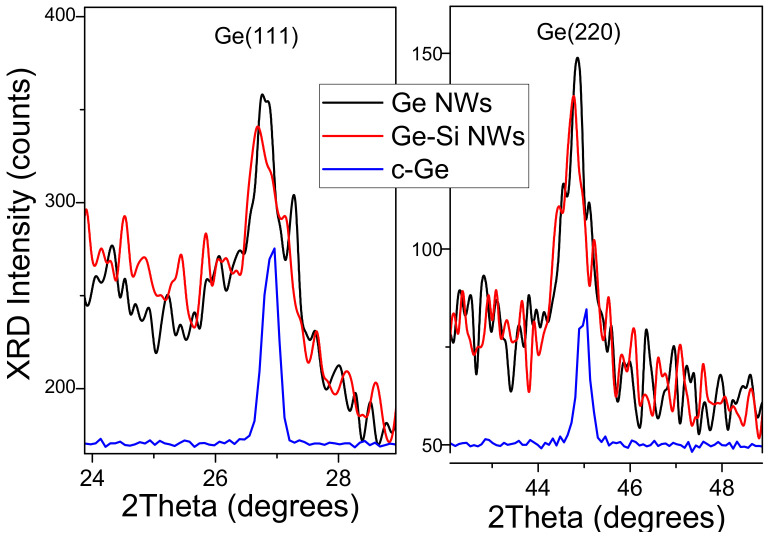
XRD angular spectra in regions of the Ge (111) and Ge (220) diffraction peaks for Ge NWs (black line), Ge-Si NWs (red line), and c-Ge (blue line).

X-ray diffraction patterns of Ge and Ge-Si NWs (Figure 5) show broadened low-intensity diffraction peaks, which confirm the crystallinity of NWs. Within the method accuracy, the peak positions for the samples of Ge and Ge-Si NWs coincide. An analysis according to the Scherrer formula of the Ge (111) and (220) line shapes shown in Figure 6 gives a coherent scattering region of 15 nm, which is in qualitative agreement with the TEM results for the sizes of nanocrystals.

### 3.5. Raman Spectroscopy

**Figure 7:**
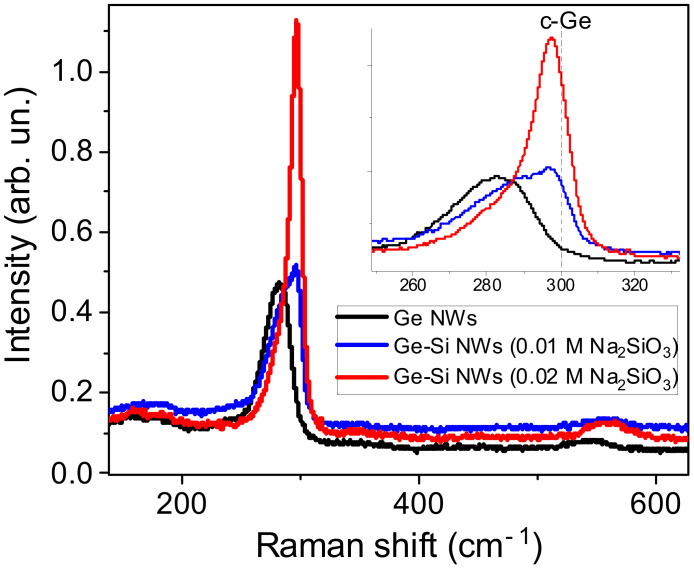
Raman spectra: Ge NWs (black line), Ge-Si NWs 0.01 M Na_2_SiO_3_ (blue curve), and Ge-Si NWs 0.02 M Na_2_SiO_3_ (red line). Inset shows the same spectra in the region of the one-phonon Raman peak for c-Ge, which is indicated by vertical dotted line.

Figure 7 shows Raman spectra of the prepared Ge NWs on Ti foil and Ge-Si NWs grown with two different concentrations of sodium silicate. A gradual shift of the germanium peak towards higher wavenumbers (from 282 to 297 cm^–1^) is observed with an increase in the sodium silicate concentration from 0.01 to 0.02 M, which indicates an increase in the crystalline fraction in agreement with the TEM data (Figure 2). One can assume two types of Ge nanocrystals in the investigated samples, i.e., small nanocrystals (Figure 2c) and whisker-like ones (Figure 2d). The latter are probably responsible for the sharp Raman signal for Ge-Si NWs prepared at higher silicate content (red line in Figure 7).

### 3.6. Electrical Properties of Ge NWs Redeposited on Chips

**Figure 8:**
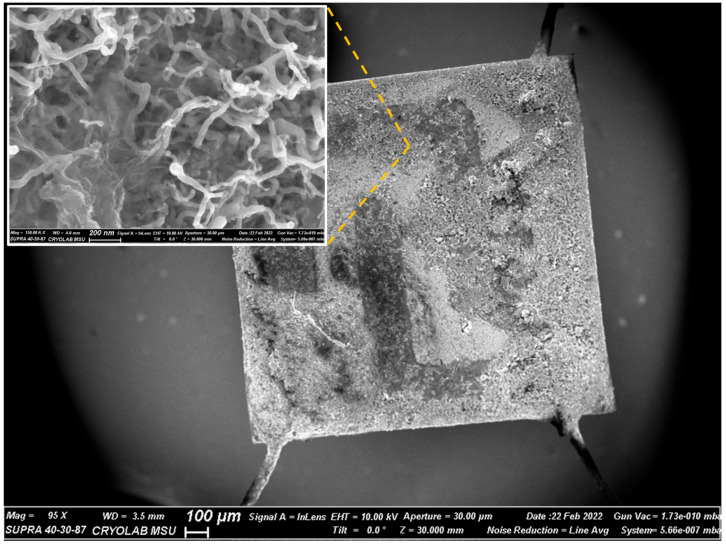
SEM image of a corundum chip with deposited Ge NWs for measurements of their electrical properties; inset shows a magnified SEM image of Ge NWs on the chip surface.

Figure 8 shows a general view of the surface of a corundum chip with a platinum electrode with Ge NWs deposited from a suspension in isopropyl alcohol. It can be seen that the threads form a fairly homogeneous layer after deposition, while their morphology and dimensions are preserved (inset in Figure 8).

**Figure 9:**
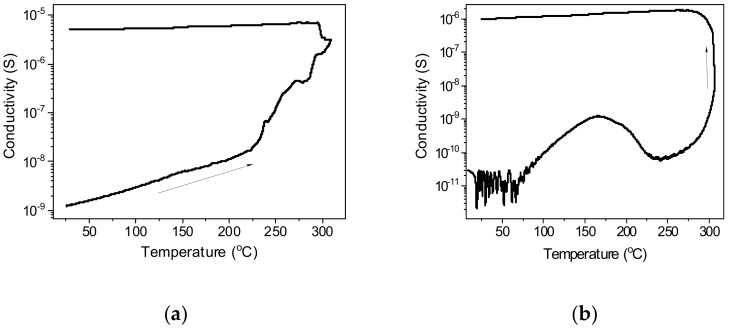
Changes in the electrical conductivity of NW films during heating in ultrahigh vacuum and subsequent cooling: (**a**) Ge NWs; (**b**) Ge-Si NWs.

**Figure 10:**
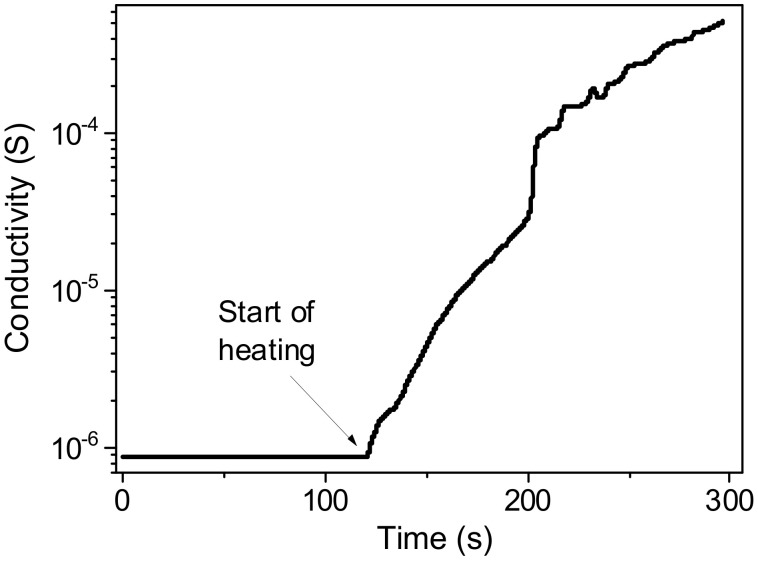
Time dependence of the electrical conductivity of Ge-Si NWs during heating at 350 °C.

It was assumed that indium, which serves as a seed for the growth of Ge NWs, as a p-type dopant, will improve the conductivity of the samples. Initially, the conductivity of the deposited films is extremely low. Upon heating in ultrahigh vacuum, after an initial increase in conductivity (Figure 9), a decrease is observed starting from a temperature of 170 °C, which we associate with the breaking of Ge-H bonds and a concomitant increase in the number of traps on the surface. Further, at a temperature of about 240 °C, this decrease is replaced by an avalanche-like increase in conductivity by 2–4 orders of magnitude, presumably due to sintering of individual NWs. This sintering process was stopped at a stage when it did not yet show signs of slowing down or completion (Figure 10). The temperature onset of the main sintering stage (280 °C) for Ge-Si sample is higher than that of Ge nanowires (240 °C). We attribute this fact to the presence of refractory SiO_2_ islets on the surface of Ge-Si nanowires. The conductivity also remained high after cooling, indicating irreversible changes in the sample. Samples obtained both with and without silicate addition show similar behavior (Figure 9a,b).

**Figure 11:**
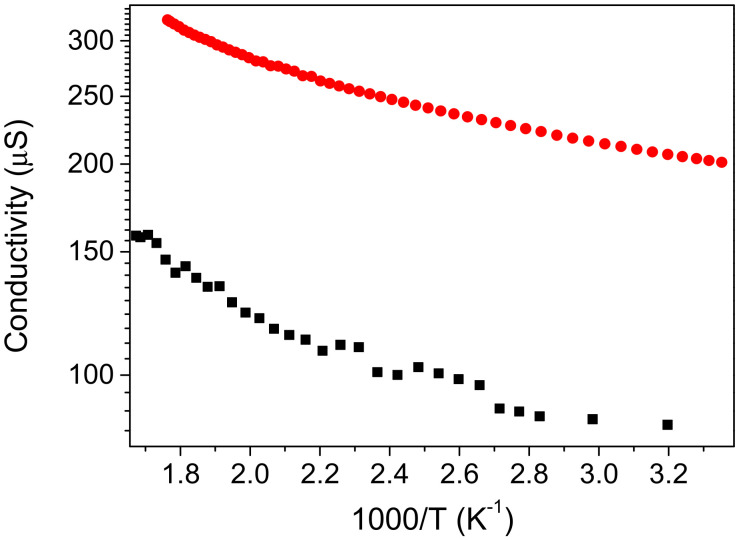
Temperature dependences of the conductivity of films of Ge NWs (black squares) and Ge-Si NWs (red circles) after partial sintering.

Figure 11 shows temperature dependences of the conductivity of Ge and Ge-Si NWs on a corundum chip in ultrahigh vacuum. On the one hand, the dependences do not exhibit a simple activation behavior. On the other hand, the observed stretched exponential dependences are not typical for the hopping mechanism of conductivity.

**Figure 12:**
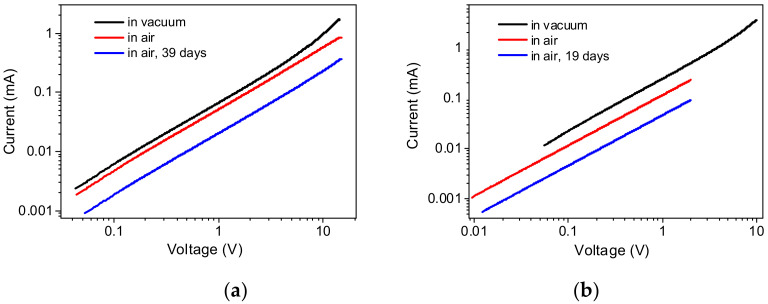
Current–voltage dependences of preheated films: (**a**) Ge NWs, (**b**) Ge-Si NWs, in vacuum and in air at room temperature.

In order to clarify the conductivity mechanism, current–voltage measurements were studied both in vacuum and in air (Figure 12). The conducting state of the samples is retained in air, but the conductivity decreases. Current–voltage dependences show some non-linearity; their shape is well-described by the equation for the current limited by the space charge:

(1) $$I=aV+bV^{m},$$

where *I* is the current, *V* is the applied voltage, *a, b, m* are fitting parameters.

It was found that samples of Ge and Ge-Si NWs stored in air exhibited a drop in the electrical conductivity. The conductivity of Ge NWs dropped by 60% in 39 days, while in the case of Ge-Si NWs, a similar decrease in conductivity occurred in 19 days.

**Figure 13:**
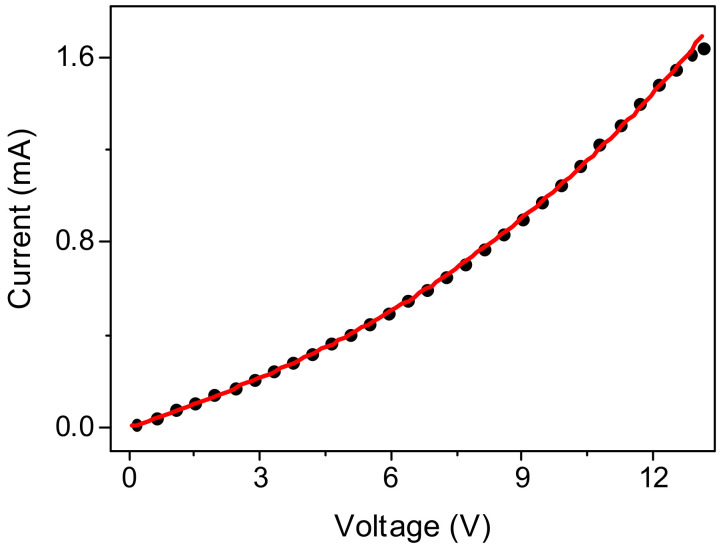
Comparison of the current–voltage dependence of a film from a Ge NW sample measured in vacuum (black circles) with an approximation by the equation for a current limited by a space charge (red line).

The parameters of the approximating curves are summarized in Table 3. For the films from the Ge-Si NW sample in air, the current–voltage dependences were linear in the studied voltage range. An example of the approximation is shown in Figure 13.

**Figure 14:**
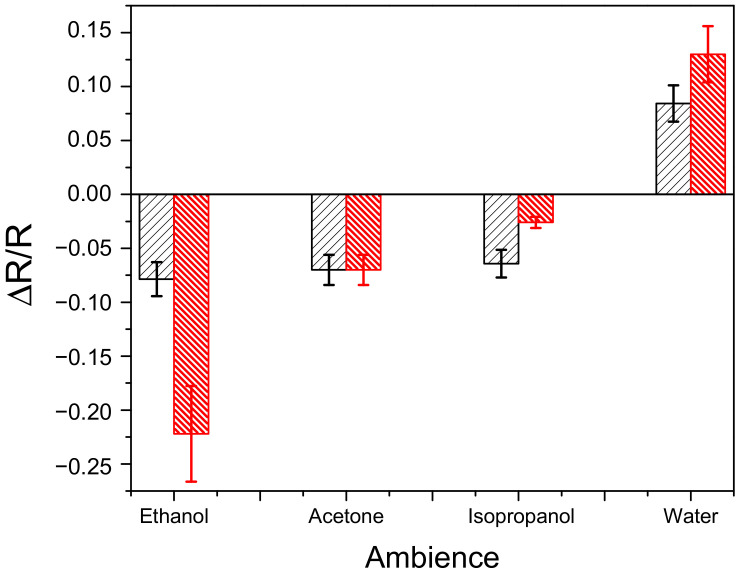
Data on the sensor properties of Ge (black columns) and Ge-Si (red columns) NW films measured as relative changes of their electrical resistance in air after admission of saturated vapors of ethanol, acetone, isopropanol, and water.

Figure 14 shows an example of the sensor response of Ge NWs deposited on corundum chips. One can see the electrical resistance of Ge NWs is highly sensitive to a molecular ambient. The samples of both Ge and Ge-Si NWs in saturated vapor of water exhibit an increase in the electrical resistance. Water is a weak donor molecule that captures holes and donates electrons. However, the main carriers in this case are holes, so the resistance increases. However, saturated vapors of organic liquids (ethanol, acetone, and isopropanol) lower the resistance of NWs, probably lowering the barriers that determine activation and conduction at room temperature. The effect of polar molecules of ethanol on the conductivity of Ge-Si NWs is much stronger relative to Ge NWs. The higher sensitivity of Ge-Si NWs in comparison with Ge ones can be explained by smaller diameters formed (see Figure 1) that should result in their higher specific surface area. In general, the sensor response of the investigated structures can be as high as 20%, which seems a good result for the potential use of Ge NWs in molecular sensorics.

## 4. Conclusions

Germanium nanowires were obtained by metal-assisted electrochemical reduction without and with the addition of sodium silicate to the electrolyte. Using electron microscopy, it was found that nanowires with the addition of sodium silicate are smaller in diameter, but are characterized by large areas of crystallinity. The crystallinity of the obtained nanowires was confirmed by X-ray diffraction and Raman spectroscopy. Using the XPS method in germanium nanowires formed with an admixture of sodium silicate, the presence of silicon in the oxide form was detected. It has been shown that the obtained nanowires can be transferred into a suspension with further deposition on various substrates with the preservation of their morphology and crystallinity. After thermal vacuum treatment of nanowire layers, they exhibit good electrical conductivity and stability of electrical characteristics. Moreover, the electrical conductivity of the nanowires is sensitive to the presence of vapors of different organic liquids and water. The sensor response, which is different for the samples prepared with and without sodium silicate, is highly dependent on the type of molecules. The observed higher sensitivity of Ge-Si nanowires to air and moisture may be linked to their diameters being smaller in comparison to the pure Ge NWs. At the same time, Ge-Si NWs are more resistant to vacuum annealing. We think that the increased sensitivity to gases is advantageous for sensing applications. Indeed, the Ge-Si sample shows increased sensitivity to water and ethanol, while sensitivity to isopropanol is decreased. Thus, the use of the same fabrication technology with different additives at varying concentrations may produce an array of sensors constituting a so-called electron nose. Potential additives include phosphate and aluminate; molybdate, leading to molybdenum blue coverage with its own sensing properties; and ferrate as a precursor of magnetite, lending its magnetic properties to the nanocomposite. The results obtained indicate new possibilities of the electrochemical method for obtaining germanium nanowires, which makes it possible to control their structural and electrical properties, which is promising for future applications in printed electronics and molecular sensorics.

## Figures and Tables

**Table 1:** Concentrations of Ge NWs and Ge-Si NWs, at %.

Sample	C	O	Ge	In	K	Na	Si	In/Ge	O/Ge	K/Ge	Na/Ge	Si/Ge
**Ge NWs**	26.8	33.6	29.7	2.4	0.9	3.6	0	0.08	1.1	0.03	0.12	0
**Ge-Si NWs**	40.0	32.7	20.0	1.5	2.1	2.1	1.6	0.08	1.6	0.11	0.11	0.08
**Ge-Si NWs after 3 min sputtering**	0	26.0	54.7	2.3	4.2	3.7	1.2	0.04	0.5	0.08	0.07	0.02

**Table 2:** Parameters of the spectra of Ge3d and In3d (E_b_, ±0.2 eV) and relative peak intensities, ±5%, of the Ge-Si NWs sample.

Surface State		Ge3d			In3d5		
		1 Ge^0^	2 GeO	3 GeO_2_	1 In^0^	2 In-Ge	3 In_2_O_3_/In(OH)_3_
**Initial**	E_b_, eV	29.4	30.5	33.0	443.9	-	445.4
	Relative peak intensity	53	23	24	40	-	60
**After 3 min sputtering**	E_b_, eV	29.4	30.5	32.5	-	444.2	-
	Relative peak intensity	75	20	5	-	100	-

**Table 3:** Parameters of fitting curves.

Sample, Conditions	a × 10^5^	b × 10^8^	m
Ge NWs, vacuum	6.4	3.4	2.52
Ge NWs, air	5.2	3.4	2.32
Ge NWs, air, 39 days	2.0	9.7	2.12
Ge-Si NWs, vacuum	22	2200	1.94
Ge-Si NWs, air	11.4	-	-
Ge-Si NWs, air, 19 days	4.6	-	-

## Data Availability

Not applicable.
